# Prognostic value of albuminuria in IgA nephropathy: insights from a real-world cohort

**DOI:** 10.1093/ckj/sfaf362

**Published:** 2025-11-22

**Authors:** Gabriel Ștefan, Adrian Zugravu, Simona Stancu

**Affiliations:** Department of Nephrology, University of Medicine and Pharmacy “Carol Davila”, Bucharest, Romania; Department of Nephrology, “Dr Carol Davila” Teaching Hospital of Nephrology, Bucharest, Romania; Department of Nephrology, University of Medicine and Pharmacy “Carol Davila”, Bucharest, Romania; Department of Nephrology, “Dr Carol Davila” Teaching Hospital of Nephrology, Bucharest, Romania; Department of Nephrology, University of Medicine and Pharmacy “Carol Davila”, Bucharest, Romania; Department of Nephrology, “Dr Carol Davila” Teaching Hospital of Nephrology, Bucharest, Romania

To the Editor,

Immunoglobulin A nephropathy (IgAN) remains a leading cause of end-stage kidney disease (ESKD), with proteinuria widely recognized as the principal prognostic marker [1]. However, while KDIGO chronic kidney disease guidelines emphasize albuminuria as a sensitive indicator of renal damage, the KDIGO IgAN recommendations continue to focus exclusively on total proteinuria [1, 2]. Notably, studies specifically addressing the prognostic value of albuminuria in IgAN are scarce, raising the question of whether albuminuria could provide additional prognostic information, particularly in patients with apparently mild proteinuria [3–5]. We therefore evaluated the relationship between albuminuria at diagnosis and the subsequent risk of ESKD in a tertiary IgAN cohort.

We retrospectively analyzed 183 adults with biopsy-proven IgAN diagnosed between 2008 and 2017 at a tertiary nephrology center in Romania. The median age at diagnosis was 43 years, 70% were male, the median estimated glomerular filtration rate (eGFR) was 38.9 mL/min/1.73 m^2^, and median proteinuria was 1.25 g/g. Only patients with an urinary albumin-to-creatinine ratio (uACR) measured from 24-h urine collection at the time of kidney biopsy were included. Participants were stratified into four uACR categories: <0.5, 0.5–1, 1–2 and >2 g/g. Follow-up extended until the onset of ESKD (initiation of kidney replacement therapy or transplantation), death or May 2024, with a median duration of 8.5 years (95% confidence interval 8.0–9.1).

**Figure 1:**
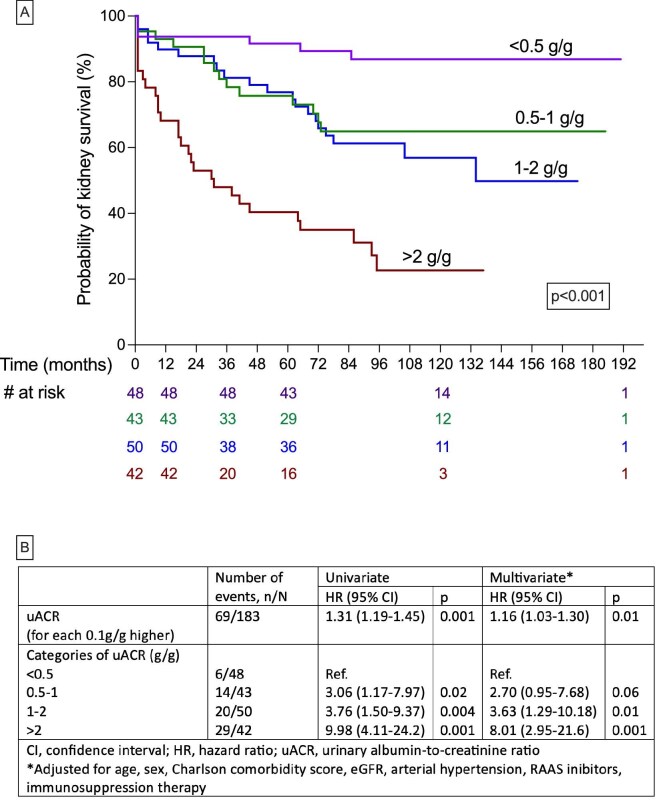
Association between uACR and survival. (**A**) Kaplan–Meier survival curves showing decreased survival with increasing uACR categories (<0.5, 0.5–1, 1–2 and >2 g/g; log-rank *P* < .001). (**B**) Cox proportional hazards analyses demonstrating that higher uACR was associated with increased mortality risk. Multivariate models were adjusted for age, sex, Charlson comorbidity score, eGFR, arterial hypertension, renin-angiotensin-aldosteron system inhibitor use and immunosuppressive therapy.

The median uACR was 0.95 g/g. Distribution across categories was 26% <0.5 g/g, 24% 0.5–1 g/g, 27% 1–2 g/g and 23% >2 g/g. Baseline demographics, comorbidity burden (Charlson index), hypertension prevalence and use of renin–angiotensin system inhibitors or immunosuppressive therapy were similar among groups. However, baseline renal function differed significantly: patients with uACR <0.5 g/g had a higher median eGFR (50.9 mL/min) than those with 0.5–1 g/g (37.4 mL/min), 1–2 g/g (37.9 mL/min) or >2 g/g (25.8 mL/min; *P* = .001). During follow-up, 69 patients (38%) reached ESKD. The mean kidney survival for the cohort was 10.5 years (95% confidence interval 9.5–11.5), with survival probabilities of 86% at 1 year, 72% at 5 years and 59% at 10 years. The cumulative incidence of ESKD increased progressively across uACR categories: 13% in <0.5 g/g, 33% in 0.5–1 g/g, 40% in 1–2 g/g and 69% in >2 g/g (Fig. 1).

In both univariate and multivariate Cox models, higher uACR—whether analyzed continuously or categorically—was strongly and independently associated with ESKD risk (Fig. 1). Compared with the <0.5 g/g group, all higher uACR strata demonstrated incrementally greater hazards for kidney failure, underscoring a graded relationship between albuminuria severity and renal prognosis (Fig. 1).

Our findings highlight that albuminuria at diagnosis provides robust prognostic information in IgAN, even among patients with seemingly “low-grade” albuminuria (0.5–1 g/g), a subgroup often considered at minimal risk. These results challenge the assumption that proteinuria below 1 g/day confers renal safety and suggest that albuminuria, a more specific marker of glomerular injury, should be integrated into IgAN risk assessment and therapeutic decision-making. Validation in larger multicenter cohorts is warranted, but our data emphasize the need to reconsider albuminuria’s role as a key prognostic parameter in IgAN.
